# Whole-Genome Sequencing Reveals a Prolonged and Persistent Intrahospital Transmission of Corynebacterium striatum, an Emerging Multidrug-Resistant Pathogen

**DOI:** 10.1128/JCM.00683-19

**Published:** 2019-08-26

**Authors:** Xuebing Wang, Haijian Zhou, Dongke Chen, Pengcheng Du, Ruiting Lan, Xiaotong Qiu, Xuexin Hou, Zhiguo Liu, Lina Sun, Shuai Xu, Xingzhao Ji, Heqiao Li, Dan Li, Jingshan Zhang, Hui Zeng, Zhenjun Li

**Affiliations:** a State Key Laboratory of Infectious Disease Prevention and Control, Chinese Center for Disease Control and Prevention, National Institute for Communicable Disease Control and Prevention, Changping, Beijing, China; b Department of Laboratory Medicine, Beijing Hospital, National Center of Gerontology, Beijing, China; c Beijing Key Laboratory of Emerging Infectious Diseases, Institute of Infectious Diseases, Beijing Ditan Hospital, Capital Medical University, Beijing, China; d School of Biotechnology and Biomolecular Sciences, University of New South Wales, Sydney, Australia; e School of Laboratory Medicine and Life Sciences, Wenzhou Medical University, Wenzhou, China; University of Iowa College of Medicine

**Keywords:** *Corynebacterium striatum*, multidrug resistance, pulsed-field gel electrophoresis, transmission, whole-genome sequencing

## Abstract

Corynebacterium striatum is an emerging multidrug-resistant (MDR) pathogen that occurs primarily among immunocompromised and chronically ill patients. However, little is known about the genomic diversity of *C. striatum*, which contributes to its long-term persistence and transmission in hospitals. In this study, a total of 192 *C. striatum* isolates obtained from 14 September 2017 to 29 March 2018 in a hospital in Beijing, China, were analyzed by antimicrobial susceptibility testing and pulsed-field gel electrophoresis (PFGE). Whole-genome sequencing was conducted on 91 isolates. Nearly all isolates (96.3%, 183/190) were MDR. The highest resistance rate was observed for ciprofloxacin (99.0%, 190/192), followed by cefotaxime (90.6%, 174/192) and erythromycin (89.1%, 171/192). PFGE separated the 192 isolates into 79 pulsotypes, and differences in core genome single-nucleotide polymorphisms (SNPs) partitioned the 91 isolates sequenced into four clades. Isolates of the same pulsotype were identical or nearly identical at the genome level, with some exceptions. Two dominant subclones, clade 3a, and clade 4a, were responsible for the hospital-wide dissemination. Genomic analysis further revealed nine resistance genes mobilized by eight unique cassettes. PFGE and whole-genome sequencing revealed that the *C. striatum* isolates studied were the result mainly of predominant clones spreading in the hospital. *C. striatum* isolates in the hospital progressively acquired resistance to antimicrobial agents, demonstrating that isolates of *C. striatum* may adapt rapidly through the acquisition and accumulation of resistance genes and thus evolve into dominant and persistent clones. These insights will be useful for the prevention of *C. striatum* infection in hospitals.

## Abstract (precis)

Corynebacterium striatum is an emerging multidrug-resistant (MDR) pathogen that occurs primarily among immunocompromised and chronically ill patients. However, little is known about the genomic diversity of *C. striatum*, which contributes to its long-term persistence and transmission in hospitals.

## INTRODUCTION

The genus *Corynebacterium* includes pathogens and nonpathogenic environmental and saprophytic species (1). Corynebacterium striatum has historically been the second most commonly isolated *Corynebacterium* species in immunocompromised patients (2), which can cause pulmonary infections (3), septicemia (4), and osteomyelitis (5).

*C. striatum*, like other *Corynebacterium* species, has developed resistance to multiple antimicrobial agents (6–8). *C. striatum* nosocomial outbreaks that were due mainly to prolonged hospitalizations, repeated broad-spectrum antibiotic exposures, and prolonged use of invasive medical devices have been reported (3). Therefore, the outbreak potential of this pathogen highlights the necessity to identify nosocomial transmissions of the organism.

Whole-genome sequencing has been emerging as a powerful tool to track disease transmission (9) and provides high resolution in the characterization of outbreaks (10). In this study, we retrospectively investigated *C. striatum* infection and transmission in a Beijing hospital from 14 September 2017 to 29 March 2018. We used pulsed-field gel electrophoresis (PFGE) and whole-genome sequencing to elucidate the genomic relationship between isolates, and results showed that some isolates recovered in adjacent wards of the hospital were genetically indistinguishable and were likely to have remained in the hospital for some time as persistent clones. The majority of the isolates showed multidrug resistance.

## MATERIALS AND METHODS

### Ethics approval.

Patients’ demographic data (see Table S1 in the supplemental material) were extracted from the patient administration system under protocols that were reviewed and approved by the Research Ethics Committee of the Chinese Center for Disease Control and Prevention. All experiments were performed in accordance with relevant regulations.

### Bacterial identification and antimicrobial susceptibility.

*C. striatum* isolates were grown on blood agar plates (Oxoid) at 37°C in ambient air for 24 h. All isolates were first identified as *C. striatum* by matrix-assisted laser desorption ionization–time of flight (MALDI-TOF) mass spectrometry (MS) using the Vitek MS (bioMérieux, France) system. The extraction was performed by using the formic acid method (bioMérieux), and samples were analyzed with a Vitek MS MALDI-TOF mass spectrometer. The spectral data were then transferred from the Vitek MS acquisition station to the Vitek MS analysis server, and identification was performed using Myla v3.2 (bioMérieux). The 16S rRNA genes of all *C. striatum* isolates identified by MALDI-TOF MS were amplified by PCR using primers as previously described (11) and sequenced. Each 16S rRNA gene sequence was compared with those available in GenBank using the blastn program, and the species was confirmed as C. striatum with ≥99% identity.

The MICs of 12 antimicrobial agents, namely, gentamicin (0.5 to 64 mg/liter) (GEN), penicillin (0.125 to 16 mg/liter) (PEN), meropenem (0.25 to 32 mg/liter) (MEM), cefotaxime (0.25 to 32 ml/liter) (CTX), erythromycin (0.125 to 16 mg/liter) (ERY), clindamycin (0.25 to 32 mg/liter) (CLI), tetracycline (0.5 to 64 mg/liter) (TET), doxycycline (0.5 to 64 mg/liter) (DOX), linezolid (0.25 to 16 mg/liter) (LZD), rifampin (0.25 to 32 mg/liter) (RIF), ciprofloxacin (0.25 to 32 mg/liter) (CIP), and vancomycin (0.25 to 16 mg/liter) (VAN) for all isolates were determined by using microdilution in cation-adjusted Muller-Hinton broth. Antibiotic panels were obtained commercially (Meihua, Guangdong, China). Colonies of each isolates were inoculated into 5% sterile saline at an optical density with a 0.5 McFarland standard. A volume of 50 μl of the bacterial suspension was added to the Muller-Hinton broth, and 100 μl of the mixtures was added into 96-well microplates. The trays were incubated at 35°C in ambient air for 24 h before the results were determined and the susceptibility breakpoint was interpreted as recommended by the CLSI (12). Escherichia coli ATCC 25922 and Streptococcus pneumoniae ATCC 49619 were used as controls.

### PFGE.

PFGE was performed for all isolates. Chromosome digestions with SwaI (New England Biolabs, USA) were performed to determine genetic relatedness as previously described by Baio et al. (13) with the following modifications: the DNA samples were electrophoresed with pulse times from 1 to 30 s over 19 h; PFGE was carried out in 0.5× TRIS-borate-EDTA-1.0% agarose gels at 14°C with a CHEF DRIII system (Bio-Rad). The DNA banding patterns were analyzed using BioNumerics version 5.1 (Applied Maths), with optimization set at 2.0% and position tolerance set at 2.0%. Similarities among PFGE patterns were identified according to the criteria established by Tenover et al. (14). Similarity analysis of PFGE patterns was performed by calculating the Dice coefficients (15), and clustering was performed using the unweighted pair group method with average linkages (UPGMA) and BioNumerics (version 5.1).

### Whole-genome sequencing, single-nucleotide polymorphism (SNP) detection, and phylogenetic analysis.

Based on the PFGE UPGMA tree, some isolates of predominant pulsotypes and others of infrequent pulsotypes were chosen for whole-genome sequencing. Whole-genome sequencing was performed on the Illumina HiSeq X Ten platform with 150-bp paired-end sequencing, using a NEBNext Ultra DNA library prep kit for Illumina sequencing (NEB, USA). All sequencing depths exceeded 100×. Low-quality reads were filtered using readfq version 10 if they met the following criteria: (i) reads containing low-quality bases (mass value, ≤38) were over 40 bp, (ii) reads containing N bases were over 10 bp, and (iii) overlaps between reads and the adapter exceeded 15 bp. All good-quality paired-end reads were assembled into scaffolds using SOAPdenovo (16, 17).

The complete genome sequence of *C. striatum* KC-Na-01 (GenBank assembly accession number GCA_002156805.1) was used as the reference, and clean reads of sequenced isolates were mapped to the reference genome by bowtie 2 software under the default parameters (18). SNPs were then identified using SAMtools (19), and SNP positions were relative to those of the reference sequence. SNPs of low quality (read depth, <5) and those located within 5 bp of each other on the same chromosome were removed, as described in previous studies (20, 21). A phylogenetic tree based on core genome SNPs was constructed using the maximum-likelihood method with the FASTTREE 2.1.10 program. The transmission route was then reconstructed based on the emergence of different SNPs and the isolation time of each isolate in the BEAST v2.4.7 program. The antimicrobial resistance genes were detected using RGI software and the Comprehensive Antibiotic Resistance Database 3.0.2 (CARD) under default parameters (22). The insertion sequences were detected using ISfinder (23).

### Data availability.

The clean reads of the 91 isolates have been deposited in GenBank’s Sequence Read Archive under accession number SRP175392.

## RESULTS

### Epidemiological distribution of isolates in the hospital.

**FIG 1:**
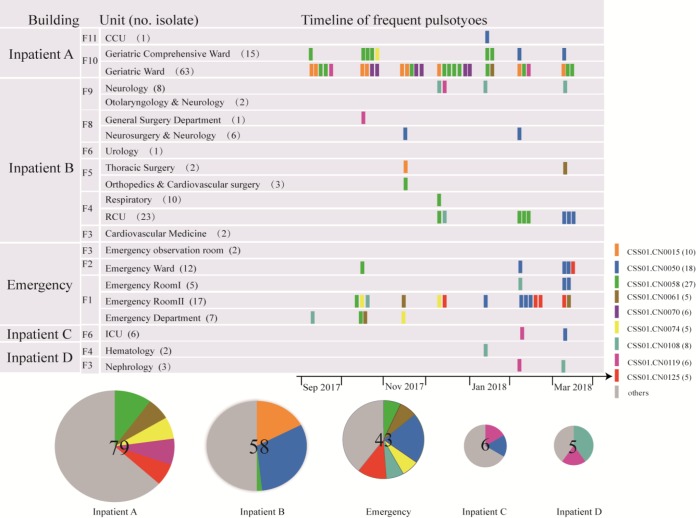
Ward distribution of Corynebacterium striatum isolates. The pies illustrate pulsotype proportions of isolates recovered from each inpatient building. Rectangles illustrate unit and isolation date distributions of 9 dominant pulsotypes of 192 Corynebacterium striatum isolates. Others include all pulsotypes except the 9 dominant ones. ICU, Intensive Care Unit; CCU, Cardiac Care Unit; RCU, Respiratory Care Unit.

A total of 192 *C. striatum* isolates were obtained from 192 patients admitted to a Beijing hospital, a 1,247-bed tertiary care hospital in Beijing, China. The isolates were obtained from 21 wards. Except for 2 urine isolates and 1 blood isolate, all other isolates were from sputum samples. Many studies have found *C. striatum* in respiratory samples (3, 24). A recent study showed that *C. striatum* can be found in a variety of anatomic sites, with the highest number from sputum (25). However, we have no explanation why our isolates were predominantly from respiratory samples. It may be that most samples came from geriatric patients who often experienced respiratory problems and sputum was one of the most common specimens for bacterial culture. Of all isolates, 63 and 15 were from the geriatric ward and geriatric comprehensive ward, respectively. Twenty-three isolates were from Respiratory Care Unit (RCU), and 10 were from the respiratory ward. The remaining were sporadically distributed among 17 other units (Fig. 1). These units were housed in 4 inpatient buildings and one emergency building. The majority of the isolates (137) were from 2 inpatient buildings, while 43 isolates were from the emergency building.

### Antimicrobial resistance profiles.

Multidrug resistance (MDR), defined as nonsusceptibility to at least one agent in three or more antimicrobial categories (8), was observed in 95.3% (183/192) of isolates. None of the isolates showed resistance against linezolid and vancomycin (see Table S2 in the supplemental material). The highest resistance rate was observed for ciprofloxacin (99.0%, 190/192), followed by cefotaxime (90.6%, 174/192), erythromycin (89.1%, 171/192), and 9 other antimicrobials to which the resistance rate was ≤88.0%.

The MDR isolates were further divided into 35 resistance profiles (Fig. S1). The most frequently encountered profile was MEM-CLI-TET-CTX-CIP-ERY-PEN (35 isolates), followed by MEM-CLI-TET-DOX-CTX-CIP-ERY-PEN and MEM-CLI-CTX-CIP-ERY-PEN (29 and 27 isolates, respectively).

### PFGE analysis.

The 192 isolates were separated into 79 distinct pulsotypes (Fig. S1). Of these, nine pulsotypes, CSS01.CN0015, CSS01.CN0050, CSS01.CN0058, CSS01.CN0061, CSS01.CN0070, CSS01.CN0074, CSS01.CN0108, CSS01.CN0119, and CSS01.CN0125, contained ≥5 isolates and were defined as predominant pulsotypes (Fig. 1). The two most frequent pulsotypes were CSS01.CN0058 and CSS01.CN0050, which contained 18 and 27 isolates, respectively.

The frequent pulsotypes provided a picture of the prevalence, persistence, and spread of *C. striatum* in the hospital (Fig. 1). The emergency building had the highest number of the frequent pulsotypes (6), while inpatient building B contained mainly three frequent types and inpatient building A contained five frequent pulsotypes. One frequent pulsotype (CSS01.CN0058) was shared by all three buildings, and the frequent pulsotypes were shared by the emergency building and inpatient building A, suggesting interbuilding spread of *C. striatum* in the hospital. By hospital unit (Fig. 1), the frequent pulsotypes persisted in the same units as well as spreading across different units. The most frequent pulsotype, CSS01.CN0058, first appeared on the geriatric floor and then appeared in the emergency department in another building. It was also isolated once from orthopedics and the cardiovascular surgery and neurology units, which were located on floors different from that of geriatric wards but within the same building. This pulsotype was isolated over the entire study period and thus persisted in the hospital on the geriatric floor. The second-most-frequent pulsotype, CSS01.CN0050, was isolated from eight units after first isolation in the neurosurgery and neurology unit, showing extensive dissemination within the hospital. All except the first isolate of CSS01.CN0050 appeared in the last 3 months of the study and was the predominate pulsotype isolated from the emergency building.

### Phylogenetic relationships of sequenced *C. striatum* isolates.

To further elucidate the diversity of *C. striatum* isolates and potential transmission events, 91 isolates, including 59 isolates belonging to the two most frequent pulsotypes, were selected for whole-genome sequencing.

**FIG 2:**
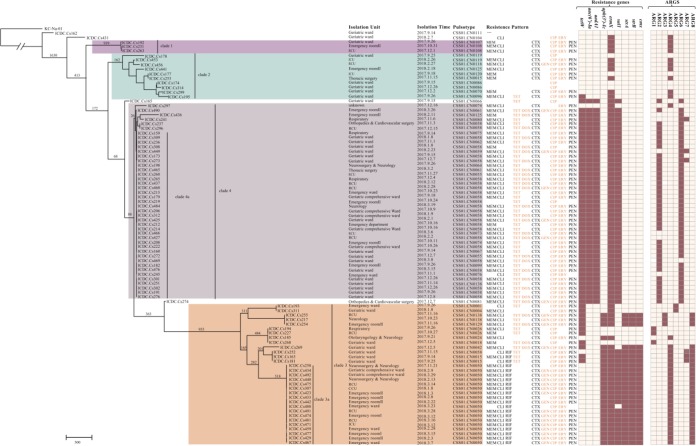
Phylogenetic relationships of 91 Corynebacterium striatum isolates based on core genome SNPs. The dark-purple-shaded part indicates isolates of clade 1, the green-shaded part indicates isolates of clade 2, the light-purple-shaded part indicates isolates of clade 3, and the orange-shaded part indicates isolates of clade 4. The information of isolate identifications, isolation unit, isolation time, pulsotypes, resistance pattern, resistance genes, and ARGs are listed to the right of the genome tree. The number on the branches corresponds to the lengths (number of SNPs) generated by the maximum-likelihood algorithm.

Genomic comparisons revealed 68,942 core genome SNPs among these isolates. Four major clades were observed (Fig. 2), among which clade 4 was the largest, with 43 isolates, compared to 31, 10, and 3 isolates in clade 3, clade 2, and clade 1, respectively. Analysis of isolates within clade 1 and clade 4 yielded SNP differences of 123 and 55 SNPs, respectively, in contrast with more than 4,000 SNPs within both clade 2 and clade 3. The genome tree showed that isolates with the same pulsotype were identical or nearly identical for the two most frequent pulsotypes. Some pulsotypes were also grouped together with the two most frequent pulsotypes, with few or no SNP differences. However, isolates of the same pulsotypes may also be located far apart on the tree: isolates in CSS01.CN0125 and CSS01.CN0015 were found in different clades.

**FIG 3:**
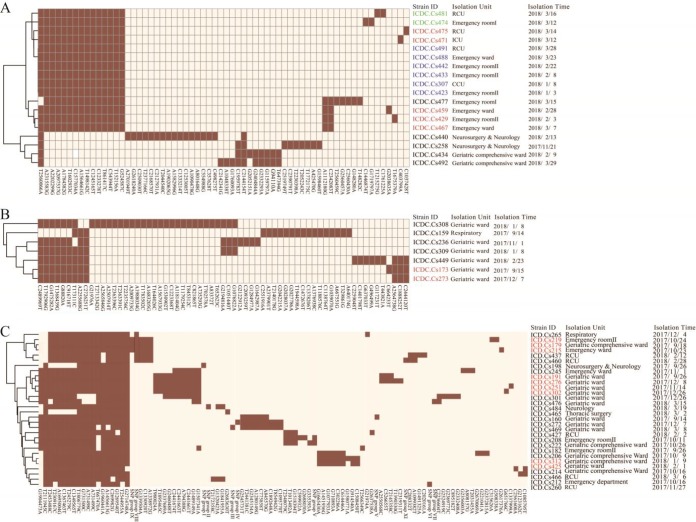
Distribution of group-specific SNPs within the two dominant clades. SNPs of each group are shown in Table S3, including SNP groups I to XI.

Based on isolation time, clade 3 and clade 4 had been circulating in the hospital for some time. We analyzed the 18 and 37 isolates from clade 3 and 4 separately to obtain higher resolution using the core genome of an individual clade and to better see direct-transmission events. The subclade tree is shown in Fig. 3 Using a cutoff of no more than 2 SNPs between two isolates for a direct-transmission event, at least eight direct-transmission events can be inferred. For example, isolates ICDC.Cs475 showed 2 SNP differences from ICDC.Cs471 (Fig. 3A), and they were obtained 2 days apart from two different units. Three isolates, ICDC.Cs459, ICDC.Cs429, and ICDC.Cs467, differed by ≤2 SNPs, and all of them were isolated from the emergency building. In addition, six isolates, ICDC.Cs491, ICDC.C488, ICDC.Cs442, ICDC.Cs433, ICDC.Cs307, and ICDC.Cs423, four of which were obtained in the emergency building, were identical in their SNPs.

While the isolates within clade 4a were more diverse than those in clade 3a, there were several discernible direct-transmission events (Fig. 3B and C). Isolates ICDC.Cs173 and ICDC.Cs273 recovered from the geriatric ward differed by 1 SNP, (Fig. 3B). Isolates ICDC.Cs179 showed 1 and 2 SNP differences from ICDC.Cs 219 and ICDC.Cs215, respectively (Fig. 3C), and all except one were isolated from the emergency building. Isolates ICDC.Cs312 and ICDC.Cs425 differed by 1 SNP, both of which were isolated from the geriatric unit.

The evolutionary relationships of the isolates also revealed patterns of the distribution of antibiotic resistance. None of the isolates within clade 1 and clade 2 showed resistance to GEN, TET, DOX, or RIF, with two exceptions. The distribution of MDR profiles within clade 4 revealed that the predominant resistance pattern was MEM-CLI-CTX-CIP-ERY-PEN, which progressively gained resistance to TET and then DOX, resulting in the resistance profiles MEM-CLI-TET-CTX-CIP-ERY-PEN and MEM-CLI-TET-DOX-CTX-CIP-ERY-PEN. GEN resistance was sporadically distributed in this clade, and all were isolated in the last 3 months. In addition, all isolates that showed resistance to RIF were located exclusively in clade 3 and were isolated within the last 3 months of the study period.

### Genomic analysis of antibiotic resistance genes.

**FIG 4:**
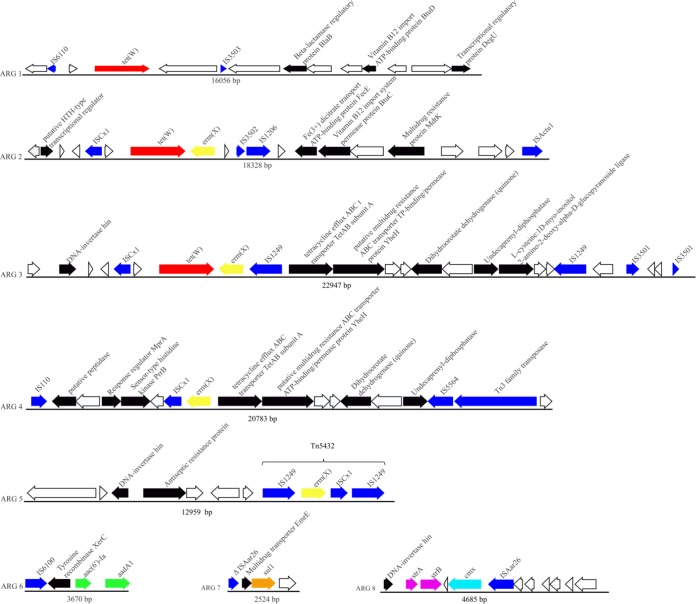
Genomic context of macrolide, lincosamide, tetracycline, and aminoglycoside resistance. Reference sequence from Tn*5432*, that of *ermX*, known to mediate macrolide and lincosamide resistance (38). Maps of mobile elements containing resistance genes are shown in ARGs, with *ermX* in yellow, *tetW* in red, aminoglycoside-modifying enzyme (AME) genes in green, insertion sequences in blue, and opening reading frames in black. Unlabeled arrows indicate hypothetical proteins.

Genomic analysis revealed nine resistance genes carried by eight unique cassettes (Fig. 2). Isolates of clade 3 harbored *aph(3′)-Ic*, *strAB*, and *cmx* genes exclusively, while clade 4 isolates harbored *aadA1* and *aac(6′)-Ia* genes. All isolates of clade 4 and nearly half of the isolates of clade 3 harbored the *tetW* gene. However, the *ermX* and *sul1* genes were present widely. Four different cassettes, namely, antibiotic resistance gene 2 (ARG2), ARG3, ARG4, and ARG5 (Fig. 4), carried the *ermX* gene, which can confer resistance to macrolides and lincosamides. Except for ARG5, the *ermX* gene of all these ARGs was bounded by an unpaired insertion sequence. All except ARG4 were distributed within a single clade (clade 3 or clade 4). ARG5 contained Tn*5432*, which carried the erythromycin resistance gene flanked by IS*1249*. ARG2 and ARG3, together with ARG1, also carried the *tetW* gene, which offers tetracycline resistance (26). Cassette ARG6 carried the *aac(6')-Ia* and *aadA1* genes and were distributed within clade 4. In addition, ARG7 carried the *sul1* gene, which can confer resistance to sulfamethoxazole, while ARG8 carried the *strAB* and *cmx* genes, which confer resistance to streptomycin and chloramphenicol, respectively. The susceptibility of our isolates to these three antimicrobials were not tested. ARG8 is carried by most of the clade 3 isolates. However, some resistance genes, such as *aph(3′)-Ic* in all isolates and *sul1* in some isolates, were not located on the cassettes, indicating that these genes were acquired independently of the cassettes.

## DISCUSSION

*C. striatum* has been reported as an emerging multidrug-resistant pathogen with the potential to cause nosocomial infections in many body sites, in particular, septicemia (4), respiratory tract infections (8), and urinary tract infections (27). In this study, we performed PFGE and whole-genome sequencing on *C. striatum* isolates from infected patients in a hospital to investigate the molecular epidemiology of this pathogen.

*C. striatum* isolates from this study showed resistance to many of the commonly used antibiotics with activity against Gram-positive organisms, such as penicillin, cefotaxime, meropenem, clindamycin, and tetracycline, to which our isolates were resistant at rates of 85.9%, 90.6%, 95.4%, 88.0%, and 58.3%, respectively; these findings are consistent with recent surveys of *C. striatum* resistance (9, 25). Nearly all isolates were MDR, which was similar to the findings from the United States and Italy (25, 28). Aminoglycosides, such as gentamicin, are used as complementary antimicrobials to treat serious infections caused by diphtheroids and showed good activity, with only 22.9% of the isolates in our study with resistance, which was similar to findings of other reports (7, 29). In addition, all isolates analyzed in our study were susceptible to vancomycin and linezolid, which was also similar to findings of other reports (1, 25).

Genome data divided the 91 isolates sequenced into four clades and seven lineages, with at least 80 SNPs supporting the branching patterns. The results suggested that multiple clones of *C. striatum* had been circulating simultaneously in the hospital. Previous studies by Gomila et al. (1) and Kang et al. (30) using multilocus sequence typing also showed different sequence types (STs) circulating in hospitals with predominant STs, suggesting that it is a common phenomenon that multiple clones circulate in the hospital environment simultaneously. Similarly, Renom et al. (31) identified the existence of nosocomial outbreaks by transmission of the same clones and the persistence of the same clones in the environment or even in patient airways for months. Our genome data provided the highest resolution to demonstrate the persistence and transmission of multiple clones in the hospital.

Meanwhile, our genome data revealed two predominant subclades circulating in different units. Clade 4a persisted mainly in geriatric units within inpatient building A across the entire study period, while clade 3a seemed to emerge and persist in the emergency building after 2018. In addition, isolates with similar genomes were repeatedly isolated from sputum samples from the same unit during the study period, suggesting that these isolates may have colonized the hospital environment for a long time before isolation (see Fig. S2 and S3 in the supplemental material).

Based on the genomic relatedness, the sources of the clones in this study may be both the community and the hospital. Clade 1 and clade 2 isolates were quite diverse, with relatively high numbers of SNPs. Many were likely to be community-acquired infections, or the clones have been circulating in the hospital environment for many years. In contrast, clade 3 and clade 4, especially subclades 3a and 4a, persisted in different units and contained very closely related isolates. Some isolates of these two subclades differed by no more than 2 SNPs, such as the ICDC.Cs273 and ICDC.Cs173 pair and the ICDC.Cs429 and ICDC.Cs467 pair. Many such cases are likely to have been hospital acquired. Infections by closely related isolates occurred in different units or even different buildings. The transmission may have occurred between patients, between health workers and patients, or through fomites or the environment. Further studies will be required to understand the route of transmission for better prevention. However, it should be noted that other hospitals may also share those clones. Closely related isolates may have been acquired through infection or colonized through a common source outside the current hospital. In the study of Nudel et al. (32), 3 cases of *C. striatum* infection with nearly identical genome sequences were identified. Investigations found that the patients had infection prior to admission and likely acquired the infection from a common outlying institution.

The genome tree showed that acquisition of drug resistance was likely to be the driving force behind *C. striatum* transmission in the hospital. Isolate ICDC.Cs162, recovered at the start of the study, was located at the base of the genome tree and was susceptible to all antimicrobial agents, and then resistance appeared among the subsequent isolates. The first acquisition was resistance to MEM, CLI, CXT, CIP, ERY, and PEN as one event, as they existed nearly across the whole-genome tree. GEN resistance first appeared in one isolate of clade 2 and sporadically occurred in clade 4. However, clustering of isolates with GEN resistance occurred within clade 3, with clade 3a likely to have acquired GEN resistance as one event. The majority of the clade 4 isolates showed resistance to TET and DOX, whereas these resistance profiles were absent in clade 3. RIF resistance occurred exclusively within clade 3. Although *C. striatum* has generally been considered to have low pathogenic potential in immunocompetent hosts, its multidrug resistance profile and intensive intrahospital dissemination underscore the importance of *C. striatum* as a nosocomial pathogen. In our study, most patients were old, which was likely to be a risk factor, as their immune systems were likely to be compromised. Therefore, *C. striatum* should be targeted in infection control and antimicrobial stewardship programs (33).

Genomic analysis further revealed diverse mobile resistance cassettes that contribute to MDR phenotypes. The study of Nudel et al. (32) identified novel vectors, including IS*3504* and IS*Cg9a*-like insertion sequences, on cassettes. In our study, resistance genes bounded by insertion sequences were different from those found by Nudel et al., indicating that our isolates were likely to have acquired and accumulated antimicrobial resistance from microorganisms found in different habitats by means of horizontal transfer of these elements. The mosaic structures of multiple mobile elements from isolates differed within clades in the genome tree, suggestive of the evolutionary consolidation of antimicrobial resistance, resulting in the generation of a multidrug-resistant bacterium. Moreover, our genome data showed that three aminoglycoside-modifying enzyme (AME) genes appeared within different clades, with *aac(6′)-Ia* and *aadA1* in clade 4 and *aph(3′)-Ic* in clade 3, which is consistent with acquisition of resistance before clade divergence and may confer a selective advantage leading to clade expansion. All cassettes were found to be unique in our study, except for Tn*5432* (ARG5), which has previously been reported in several opportunistic pathogens, such as Corynebacterium xerosis (34) and Propionibacterium acnes (35), suggesting that the resistance to erythromycin and clindamycin within *C. striatum* isolates was likely to be the consequence of interspecies horizontal transfer of Tn*5432*.

The combination of PFGE and genome data revealed extensive transmission of predominant clones between different units in the hospital. Based on PFGE data, the nine frequent pulsotypes were distributed in different units. CSS01.CN0015 and CSS01.CN0058 persisted across the entire study period, while CSS01.CCN0050 emerged in the second half of the study period. A previous study by Verroken et al. (36) using PFGE showed that *C. striatum* caused outbreaks as an opportunistic nosocomial pathogen in patients hospitalized for a long period. The study of Wang et al. (37) also found that three pulsotypes spread across different departments in a hospital and persisted over the 14-month study period, showing similar extensive transmissions. However, it should be cautioned that pulsotypes may not be homogenous, as was found in our study of CSS01.CN0015. Whole-genome sequencing provided the definite evidence of genetic relatedness of the isolates and persistence and potential transmission of the same types within the hospital.

### Conclusions.

Our results demonstrated that *C. striatum* has the potential to progressively acquire resistance to antimicrobial agents and thus evolve into predominant clones to persist and circulate within a hospital. Our study also shed light on intrahospital transmission. These findings will be useful for the prevention of *C. striatum* nosocomial infections.

## Supplementary Material

Supplemental file 1
