# Supplementary material for: Whole-Genome Sequencing Reveals a Prolonged and Persistent Intrahospital Transmission of Corynebacterium striatum, an Emerging Multidrug-Resistant Pathogen
Source: J Clin Microbiol. 2019 Aug 26;57(9):e00683-19. doi: 10.1128/JCM.00683-19 (PMC6711910; doi:10.1128/JCM.00683-19)
Supplement: Supplemental file 1 [file JCM.00683-19-s0001.pdf]

Table S1. List of *Corynebacterium striatum* strains analyzed in this study

No. of strain	Gender/age	Sample type	Isolation time	Isolation unit
ICDC.Cs158	M/90	Sputum	September 14, 2017	Geriatric comprehensive ward
ICDC.Cs159	M/81	Sputum	September 14, 2017	Respiratory
ICDC.Cs160	M/96	Sputum	September 14, 2017	Geriatric ward
ICDC.Cs161	M/93	Sputum	September 19, 2017	Geriatric comprehensive ward
ICDC.Cs162	M/93	Sputum	September 14, 2017	Geriatric ward
ICDC.Cs163	M/98	Sputum	September 14, 2017	Geriatric ward
ICDC.Cs164	M/83	Sputum	September 15, 2017	Respiratory
ICDC.Cs165	M/93	Sputum	September 15, 2017	Geriatric ward
ICDC.Cs173	M/94	Sputum	September 15, 2017	Geriatric ward
ICDC.Cs174	F/97	Sputum	September 15, 2017	Geriatric ward
ICDC.Cs177	M/82	Sputum	September 18, 2017	ICU
ICDC.Cs178	M/87	Sputum	September 25, 2017	Geriatric ward
ICDC.Cs179	F/87	Sputum	September 18, 2017	Geriatric comprehensive ward
ICDC.Cs180	M/86	Sputum	September 18, 2017	Neurology
ICDC.Cs181	M/79	Sputum	September 25, 2017	Geriatric ward
ICDC.Cs182	F/96	Sputum	September 26, 2017	Emergency room Ⅱ
ICDC.Cs185	F/62	Sputum	September 21, 2017	Otolaryngology & Neurology
ICDC.Cs186	M/90	Sputum	September 21, 2017	Emergency ward
ICDC.Cs187	M/100	Sputum	September 21, 2017	Geriatric ward
ICDC.Cs188	M/95	Sputum	September 21, 2017	Geriatric ward
ICDC.Cs189	M/81	Sputum	September 26, 2017	Emergency ward
ICDC.Cs190	F/72	Sputum	September 26, 2017	Respiratory
ICDC.Cs191	M/95	Sputum	September 26, 2017	Geriatric ward
ICDC.Cs192	M/88	Sputum	September 26, 2017	Geriatric ward
ICDC.Cs193	M/89	Sputum	September 26, 2017	Emergency ward
ICDC.Cs194	M/81	Sputum	September 26, 2017	Respiratory
ICDC.Cs195	M/99	Sputum	September 26, 2017	Geriatric ward
ICDC.Cs196	M/92	Sputum	September 26, 2017	Geriatric ward
ICDC.Cs197	M/89	Sputum	September 26, 2017	Emergency ward
ICDC.Cs198	F/83	Sputum	September 26, 2017	Neurosurgery & Neurology
ICDC.Cs199	F/76	Sputum	September 27, 2017	Emergency department
ICDC.Cs200	M/90	Sputum	October 18, 2017	Geriatric ward
ICDC.Cs202	F/62	Sputum	October 11, 2017	Emergency department
ICDC.Cs203	M/73	Sputum	October 9, 2017	Emergency department
ICDC.Cs204	F/95	Sputum	October 9, 2017	Geriatric ward
ICDC.Cs205	F/98	Sputum	October 9, 2017	Geriatric ward
ICDC.Cs206	F/96	Sputum	October 9, 2017	Geriatric comprehensive ward
ICDC.Cs208	M/86	Sputum	October 11, 2017	Emergency room Ⅱ
ICDC.Cs209	M/91	Sputum	October 11, 2017	Geriatric ward
ICDC.Cs210	F/102	Sputum	October 13, 2017	Geriatric comprehensive ward

ICDC.Cs211	M/101	Sputum	October 14, 2017	Geriatric ward
ICDC.Cs212	M/82	Sputum	October 16, 2017	Emergency department
ICDC.Cs214	F/93	Sputum	October 16, 2017	Geriatric comprehensive ward
ICDC.Cs215	M/83	Sputum	October 23, 2017	Emergency ward
ICDC.Cs216	M/94	Sputum	October 24, 2017	Geriatric ward
ICDC.Cs217	M/86	Sputum	October 23, 2017	Neurology
ICDC.Cs218	M/107	Sputum	October 23, 2017	Geriatric ward
ICDC.Cs219	F/87	Sputum	October 24, 2017	Emergency room II
ICDC.Cs220	M/87	Sputum	October 25, 2017	Geriatric ward
ICDC.Cs222	M/89	Sputum	October 26, 2017	Geriatric comprehensive ward
ICDC.Cs223	M/93	Sputum	October 26, 2017	Respiratory
ICDC.Cs224	M/91	Sputum	October 26, 2017	RCU
ICDC.Cs225	M/87	Sputum	October 26, 2017	General surgery department
ICDC.Cs226	F/90	Sputum	October 26, 2017	Emergency observation room
ICDC.Cs227	F/95	Sputum	October 27, 2017	RCU
ICDC.Cs228	M/67	Sputum	October 3, 2017	Urology
ICDC.Cs229	M/90	Sputum	October 3, 2017	Geriatric ward
ICDC.Cs230	M/80	Sputum	October 31, 2017	RCU
ICDC.Cs231	M/62	Sputum	October 31, 2017	Emergency room II
ICDC.Cs232	M/90	Sputum	October 31, 2017	Geriatric comprehensive ward
ICDC.Cs233	F/80	Sputum	October 31, 2017	Cardiovascular Medicine
ICDC.Cs234	M/79	Sputum	November 1, 2017	Geriatric ward
ICDC.Cs235	M/94	Sputum	November 2, 2017	Geriatric ward
ICDC.Cs236	M/95	Sputum	November 1, 2017	Geriatric ward
ICDC.Cs237	M/81	Sputum	November 3, 2017	Orthopedics & cardiovascular surgery
ICDC.Cs238	M/85	Sputum	November 3, 2017	Geriatric comprehensive ward
ICDC.Cs239	M/77	Sputum	November 6, 2017	Respiratory
ICDC.Cs240	M/45	Sputum	November 7, 2017	Otolaryngology & Neurology
ICDC.Cs241	M/80	Sputum	November 6, 2017	Respiratory
ICDC.Cs242	M/56	Sputum	November 7, 2017	Respiratory
ICDC.Cs243	M/96	Sputum	November 9, 2017	Geriatric ward
ICDC.Cs244	M/99	Sputum	November 9, 2017	Geriatric ward
ICDC.Cs245	M/94	Sputum	November 1, 2017	Emergency ward
ICDC.Cs246	M/81	Sputum	November 1, 2017	RCU
ICDC.Cs247	M/91	Sputum	November 13, 2017	Geriatric ward
ICDC.Cs248	M/91	Sputum	November 13, 2017	Geriatric ward
ICDC.Cs249	M/72	Sputum	November 13, 2017	Emergency room II
ICDC.Cs250	M/83	Sputum	November 14, 2017	ICU
ICDC.Cs251	F/101	Sputum	November 14, 2017	Geriatric ward
ICDC.Cs252	M/98	Sputum	November 15, 2017	Geriatric ward
ICDC.Cs253	M/88	Sputum	November 15, 2017	Thoracic surgery
ICDC.Cs254	M/60	Sputum	November 16, 2017	Emergency room II
ICDC.Cs255	M/90	Sputum	November 16, 2017	RCU

ICDC.Cs256	F/77	Sputum	November 16, 2017	Emergency department
ICDC.Cs257	M/85	Sputum	November 2, 2017	Emergency department
ICDC.Cs258	F/83	Sputum	November 21, 2017	Neurosurgery & Neurology
ICDC.Cs259	M/96	Sputum	November 24, 2017	Geriatric ward
ICDC.Cs260	M/80	Sputum	November 27, 2017	RCU
ICDC.Cs261	F/80	Sputum	December 2, 2017	Neurology
ICDC.Cs262	M/74	Sputum	December 2, 2017	Neurology
ICDC.Cs263	F/80	Sputum	December 1, 2017	RCU
ICDC.Cs264	M/89	Sputum	December 4, 2017	Emergency observation room
ICDC.Cs265	M/63	Sputum	December 4, 2017	Respiratory
ICDC.Cs266	M/90	Sputum	December 4, 2017	Geriatric ward
ICDC.Cs267	M/87	Sputum	December 4, 2017	Geriatric ward
ICDC.Cs268	M/81	Sputum	December 5, 2017	Geriatric ward
ICDC.Cs269	M/90	Sputum	December 5, 2017	Geriatric ward
ICDC.Cs270	M/81	Sputum	December 6, 2017	Neurosurgery & Neurology
ICDC.Cs271	F/87	Sputum	December 11, 2017	Emergency room Ⅱ
ICDC.Cs272	F/94	Sputum	December 7, 2017	Geriatric ward
ICDC.Cs273	M/94	Sputum	December 7, 2017	Geriatric ward
ICDC.Cs274	M/54	Sputum	December 7, 2017	Orthopedics & cardiovascular surgery
ICDC.Cs275	M/93	Sputum	December 8, 2017	Emergency room Ⅱ
ICDC.Cs276	F/94	Sputum	December 8, 2017	Geriatric ward
ICDC.Cs277	F/80	Sputum	December 11, 2017	Cardiovascular Medicine
ICDC.Cs278	F/80	Sputum	December 11, 2017	Geriatric comprehensive ward
ICDC.Cs296	M/75	Sputum	December 15, 2017	RCU
ICDC.Cs297	M/86	Sputum	December 16, 2017	Unknown
ICDC.Cs298	F/95	Sputum	December 19, 2017	Geriatric ward
ICDC.Cs299	M/88	Sputum	December 2, 2017	Geriatric ward
ICDC.Cs300	M/76	Sputum	December 26, 2017	Orthopedics & cardiovascular surgery
ICDC.Cs301	M/95	Sputum	December 26, 2017	Geriatric ward
ICDC.Cs302	M/95	Sputum	December 26, 2017	Geriatric ward
ICDC.Cs303	F/73	Sputum	December 27, 2017	Neurology
ICDC.Cs304	M/98	Sputum	December 28, 2017	Geriatric ward
ICDC.Cs305	F/90	Sputum	January 4, 2018	Emergency department
ICDC.Cs306	F/83	Sputum	January 4, 2018	ICU
ICDC.Cs307	M/44	Sputum	January 8, 2018	CCU
ICDC.Cs308	M/85	Sputum	January 8, 2018	Geriatric ward
ICDC.Cs309	M/96	Sputum	January 8, 2018	Geriatric ward
ICDC.Cs310	M/84	Sputum	January 8, 2018	Geriatric comprehensive ward
ICDC.Cs311	F/95	Sputum	January 8, 2018	Geriatric ward
ICDC.Cs312	F/103	Sputum	January 9, 2018	Geriatric comprehensive ward
ICDC.Cs313	M/94	Sputum	January 9, 2018	Respiratory
ICDC.Cs314	M/102	Urine	December 26, 2017	Geriatric ward

ICDC.Cs421	M/101	Sputum	January 29, 2018	Geriatric ward
ICDC.Cs422	M/81	Sputum	January 3, 2018	Neurology
ICDC.Cs423	M/83	Sputum	January 3, 2018	Emergency room II
ICDC.Cs424	M/70	Sputum	January 31, 2018	Hematology
ICDC.Cs425	F/99	Sputum	February 1, 2018	Geriatric ward
ICDC.Cs426	M/95	Sputum	February 1, 2018	Geriatric ward
ICDC.Cs427	M/86	Sputum	February 2, 2018	RCU
ICDC.Cs428	M/70	Sputum	February 2, 2018	Hematology
ICDC.Cs429	F/90	Sputum	February 3, 2018	Emergency room II
ICDC.Cs431	M/86	Urine	February 7, 2018	Geriatric ward
ICDC.Cs432	M/90	Sputum	February 6, 2018	Emergency ward
ICDC.Cs433	M/83	Sputum	February 8, 2018	Emergency room II
ICDC.Cs434	M/78	Sputum	February 9, 2018	Geriatric comprehensive ward
ICDC.Cs435	F/84	Sputum	February 1, 2018	RCU
ICDC.Cs436	M/93	Sputum	February 11, 2018	Emergency room II
ICDC.Cs437	F/81	Sputum	February 12, 2018	RCU
ICDC.Cs438	F/98	Sputum	February 12, 2018	RCU
ICDC.Cs440	F/62	Sputum	February 13, 2018	Neurosurgery & Neurology
ICDC.Cs441	M/79	Blood	February 18, 2018	Emergency room II
ICDC.Cs442	M/82	Sputum	February 22, 2018	Emergency room II
ICDC.Cs444	M/79	Sputum	February 23, 2018	RCU
ICDC.Cs445	M/83	Sputum	February 23, 2018	ICU
ICDC.Cs446	F/102	Sputum	February 23, 2018	Geriatric ward
ICDC.Cs447	M/105	Sputum	February 23, 2018	Geriatric ward
ICDC.Cs448	M/88	Sputum	February 23, 2018	RCU
ICDC.Cs449	M/94	Sputum	February 23, 2018	Geriatric ward
ICDC.Cs450	M/85	Sputum	February 24, 2018	RCU
ICDC.Cs451	F/89	Sputum	February 26, 2018	Nephrology
ICDC.Cs452	M/63	Sputum	February 26, 2018	RCU
ICDC.Cs453	M/99	Sputum	February 26, 2018	ICU
ICDC.Cs454	F/83	Sputum	February 27, 2018	Emergency room I
ICDC.Cs455	M/81	Sputum	February 27, 2018	Geriatric ward
ICDC.Cs456	M/92	Sputum	February 27, 2018	RCU
ICDC.Cs457	M/98	Sputum	February 27, 2018	Geriatric ward
ICDC.Cs458	M/81	Sputum	February 27, 2018	Neurosurgery & Neurology
ICDC.Cs459	M/85	Sputum	February 28, 2018	Emergency ward
ICDC.Cs460	M/66	Sputum	February 28, 2018	RCU
ICDC.Cs461	M/88	Sputum	February 28, 2018	Geriatric ward
ICDC.Cs462	F/84	Sputum	February 28, 2018	Emergency ward
ICDC.Cs463	M/32	Sputum	March 1, 2018	Neurology
ICDC.Cs464	M/97	Sputum	March 2, 2018	Geriatric comprehensive ward
ICDC.Cs465	M/65	Sputum	March 2, 2018	Thoracic surgery
ICDC.Cs466	M/86	Sputum	March 6, 2018	RCU
ICDC.Cs467	M/63	Sputum	March 7, 2018	Emergency ward

ICDC.Cs468	M/97	Sputum	March 8, 2018	Geriatric ward
ICDC.Cs469	F/94	Sputum	March 8, 2018	Geriatric ward
ICDC.Cs470	M/83	Sputum	March 9, 2018	Emergency room Ⅱ
ICDC.Cs471	M/51	Sputum	March 12, 2018	ICU
ICDC.Cs472	M/65	Sputum	March 12, 2018	Neurosurgery & Neurology
ICDC.Cs473	M/85	Sputum	March 12, 2018	Emergency room Ⅰ
ICDC.Cs474	M/89	Sputum	March 12, 2018	Emergency room Ⅰ
ICDC.Cs475	M/66	Sputum	March 14, 2018	RCU
ICDC.Cs476	M/95	Sputum	March 15, 2018	Geriatric ward
ICDC.Cs477	M/87	Sputum	March 15, 2018	Emergency room Ⅰ
ICDC.Cs478	M/81	Sputum	March 15, 2018	Geriatric ward
ICDC.Cs479	M/91	Sputum	March 16, 2018	Emergency ward
ICDC.Cs481	M/79	Sputum	March 16, 2018	RCU
ICDC.Cs482	F/96	Sputum	March 16, 2018	Nephrology
ICDC.Cs483	M/94	Sputum	March 17, 2018	Emergency room Ⅰ
ICDC.Cs484	F/86	Sputum	March 19, 2018	Neurology
ICDC.Cs486	F/78	Sputum	March 26, 2018	Nephrology
ICDC.Cs487	M/89	Sputum	March 26, 2018	Emergency room Ⅱ
ICDC.Cs488	M/89	Sputum	March 23, 2018	Emergency ward
ICDC.Cs489	M/72	Sputum	March 26, 2018	RCU
ICDC.Cs490	M/75	Sputum	March 26, 2018	Emergency room Ⅱ
ICDC.Cs491	M/90	Sputum	March 28, 2018	RCU
ICDC.Cs492	M/78	Sputum	March 29, 2018	Geriatric comprehensive ward

* M, male; F, female.

Table S2. Susceptibility of 192 *Corynebacterium striatum* isolates to 12 antimicrobial agents

			Breakpoint				
**Antimicrobial agent**	**Range(mg/L)**	**MIC90**	**S**	**I**	**R**	**Number**	**R（%）**
**Gentamicin**	≤0.5->64	>16	≤4	8	≥16	192	44(22.9)
**Penicillin**	≤0.125->16	>16	≤0.12	0.25-2	≥4	192	165(85.9)
**Meropenem**	≤0.25->32	>32	≤0.25	0.5	≥1	192	155(80.7)
**Cefotaxime**	≤0.25->32	>32	≤1	2	≥4	192	174(90.6)
**Erythromycin**	≤0.125->16	>16	≤0.5	1	≥2	192	171(89.1)
**Clindamycin**	≤0.25->32	>32	≤0.5	1-2	≥4	192	169(88.0)
**Tetracycline**	≤0.5->64	>64	≤4	8	≥16	192	112(58.3)
**Doxycycline**	≤0.5->64	<64	≤4	8	≥16	192	54(28.1)
**Linezolid**	≤0.25->1	<0.5	≤2			192	0
**Rifampicin**	≤0.25->32	>32	≤1	2	≥4	192	33(17.2)
**Ciprofloxacin**	0.5->32	<32	≤1	2	≥4	192	190(99.0)
**Vancomycin**	0.25-1	<0.5	≤2			192	0

Isolates were classified as resistant (R), intermediate (I), or susceptible (S), according to criteria defined by CLSI;

MIC: minimum inhibitory concentration;

MIC90: MIC that inhibits 90% of the isolates.

Table S3. SNPs of each group

SNPs							
**SNP group I**	**SNP group II**	**SNP group III**	**SNP group IV**	**SNP group V**	**SNP group VI**	**SNP group VII**	**SNP group VIII**
C2666019G	T2705128C	G2320353A	G2114676A	G2316592A	G2604833A	C2444120T	G2731612T
A2562006C	T2668344C	T2320343C	C1807516T	G2127703C	C2546333T	T1742136G	G2703409T
G2334056T	T2596979C	G1595271C	C1682159T	G2008087A	A2128094G	C1376277T	C2651084T
A2333843C	A2379901T	G482239A	C1623962T	G1884728T	C1605131T	T476070C	C2583093A
A2317722C	A2354201G	G917577A	T811006G	G1582709T	C90891T	G188145A	C2435102A
T2316220G	T2334612C	G2398035A	A334895G	C583232T	G296343A	T188146G	G2111942A
G2274464T	A2275392G		C743798T	C1381074T			C1844444T
T2260894G	A2270606G						G1816184T
C2239919A	A2128120G						C1118187A
A2233814C	A2068833G						C841103A
T2122181G	T2053675C						C803948A
G2118297T	C1900683T						G517004A
T2038336G	T1900681C						G644000T
A2034751C	G1884840A						
A2018464C	A1851598G						
C1975931T	T1830499C						
G1872251T	T1780856C						
A1815739C	A1666092G						
A1766325C	T1580911C						
A1739538C	C1563786T						
T1678410C	A1466168G						
A1675047C	A1452569G						
C1603656G	A1421703G						
C1584366G	A1395533G						
T1547212G	A1220905G						
A1509109C	T1183556C						
T1484853G	A1167477G						
G1483939A	A1074423G						
G1482232C	T1018667C						
T1407592G	A745793G						
A1391956C	A738624G						
G1369204T	T706809C						
C1287935T	G681618T						
G1210632A	T591812C						
T1049983G	A445713G						
G1041327A	T407066C						
T1033085G	A372067G						
C1012568T	A300689G						
T978212G	T275158C						

T963826G	T187409C
A869246G	C188276A
A843325G	T2724601C
A776243G	
T747479G	
A733766C	
G443447C	
T419300C	
T333513G	
G397112A	
A2698002C	

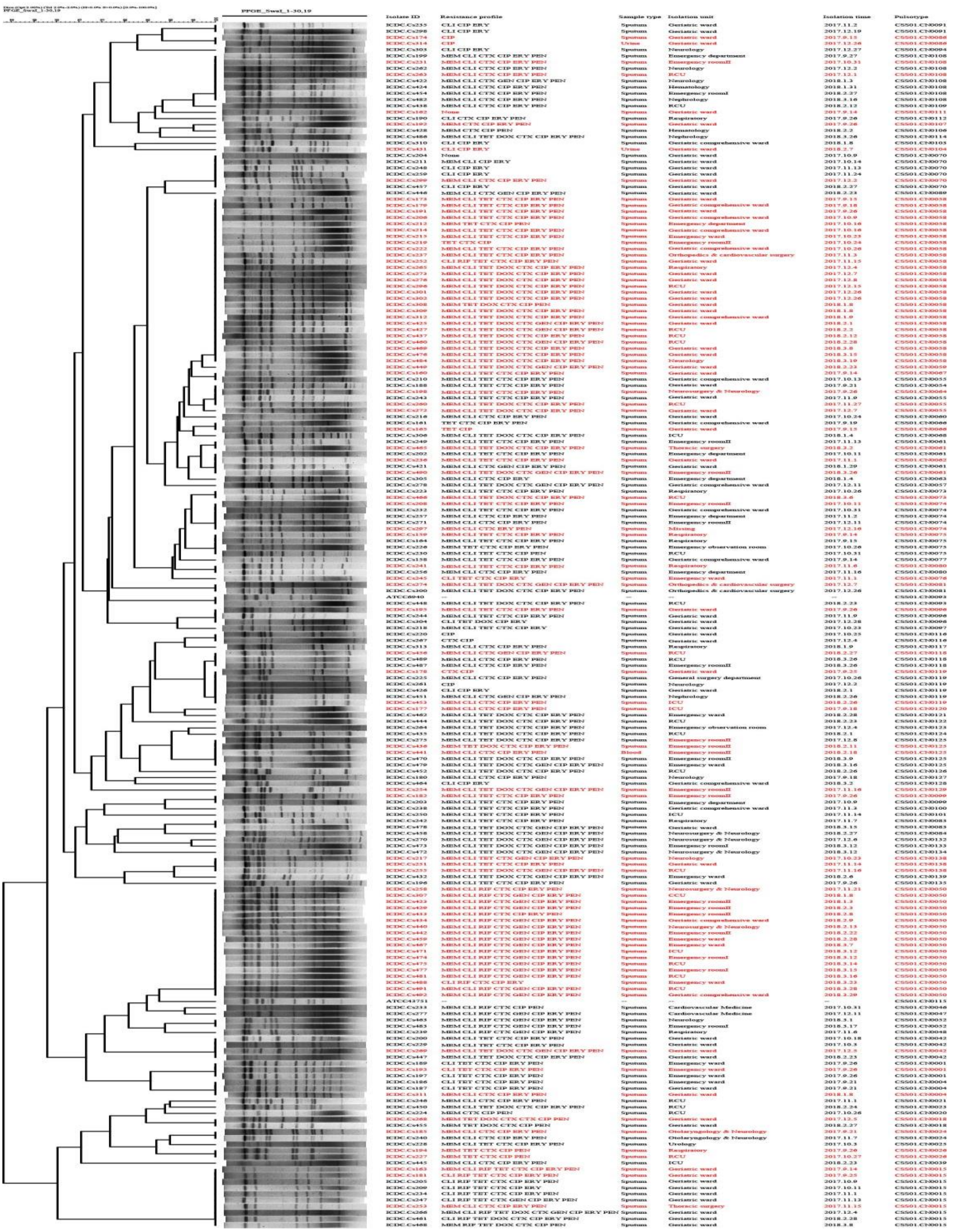

Fig. S1. Dendrogram of the 192 isolates using the unweighted pair group method with arithmetic based on pulsotype field gel electrophoresis (PFGE) patterns of the isolates. The isolate ID, resistance profile, sample type, isolation unit, isolation date and pulsotype are shown on the right of the gendrogram. Isolates chosen for whole genome sequencing were marked in red. GEN, gentamicin; PEN, penicillin; MEM, meropenem; CTX, cefotaxime; ERY, erythromycin; CLI, clindamycin; TET, tetracycline; DOX, doxycycline; RIF, rifampicin; CIP, ciprofloxacin. None, susceptible to all compounds.

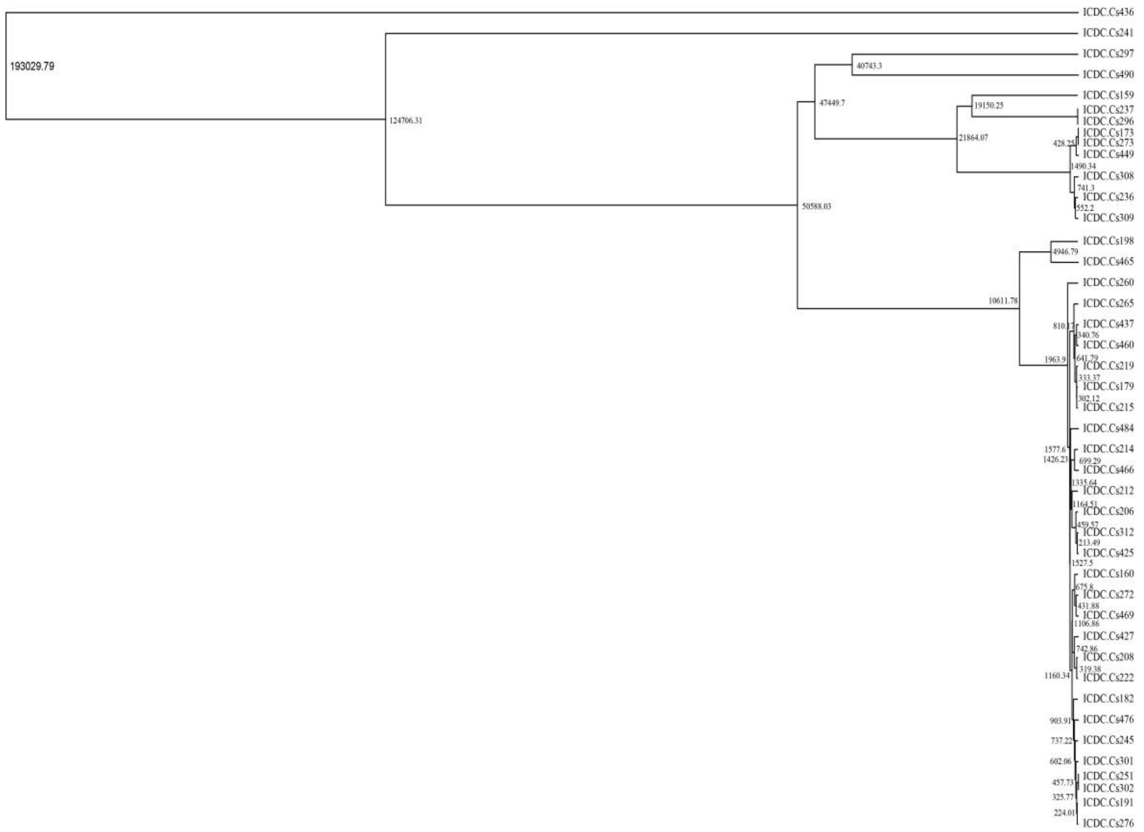

Fig. S2. Bayesian estimation of divergence times of clade 3 (days). Numbers on the tree indicate evolutionary time before the last day of isolation time for each branch.

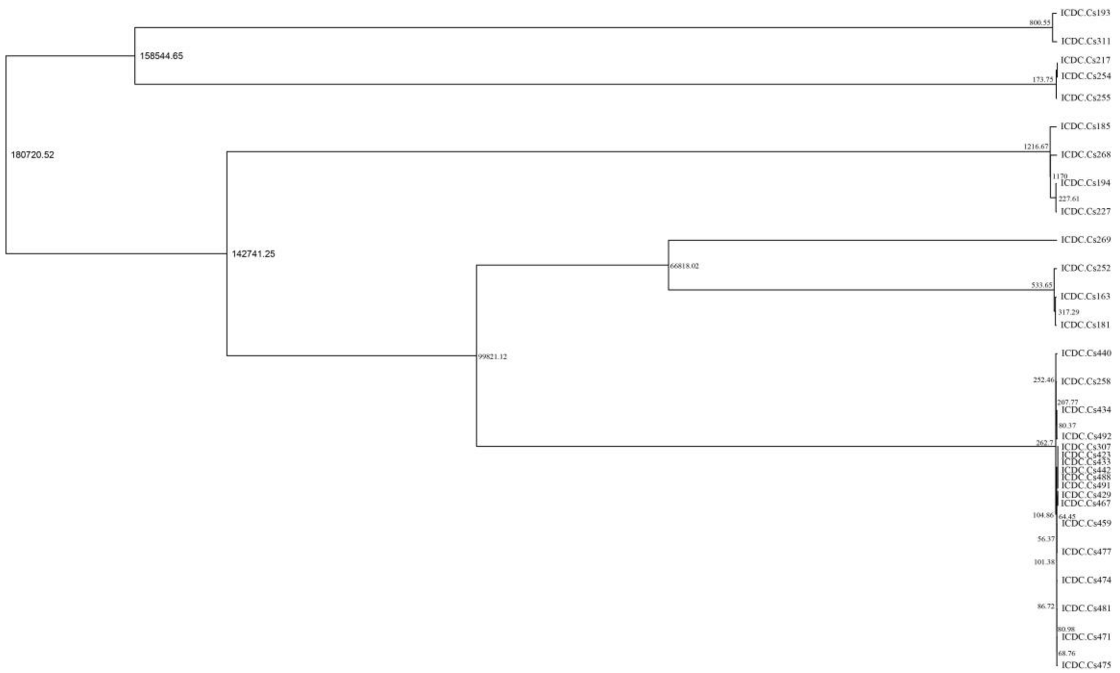

Fig. S3. Bayesian estimation of divergence times of clade 4 (days). Numbers on the tree indicate evolutionary time before the last day of isolation time for each branch.
